# Observational Study Regarding Possible Side Effects of Miniscrew-Assisted Rapid Palatal Expander (MARPE) with or without the Use of Corticopuncture Therapy

**DOI:** 10.3390/biology10030187

**Published:** 2021-03-03

**Authors:** Eugen Silviu Bud, Cristina Ioana Bică, Mariana Păcurar, Petru Vaida, Alexandru Vlasa, Krisztina Martha, Anamaria Bud

**Affiliations:** 1 Faculty of Dental Medicine, University of Medicine and Pharmacy, Science and Technology George Emil Palade Târgu-Mureș, 38 Gheorghe Marinescu Street, 540139 Târgu Mureș, Romania; Eugen.bud@umfst.ro (E.S.B.); cristina.bica@umfst.ro (C.I.B.); Mariana.pacurar@umfst.ro (M.P.); krisztina.martha@umfst.ro (K.M.); Anamaria.bud@umfst.ro (A.B.); 2 Dudley Group of Hospitals NHS Foundation Trust, Birmingham B18 7QH, UK; Petru.vaida@nhr.net

**Keywords:** MARPE, orthodontic miniscrew, palatal expansion, CBCT, MSE

## Abstract

**Simple Summary:**

In this observational study, we evaluated possible complications at the skeletal and dentoalveolar level after palatal split using miniscrew-assisted rapid palatal expansion (MARPE) associated or not with corticopuncture (CP) therapy. The study included 27 patients with maxillary transverse deficiency and unilateral or bilateral cross-bite. Skeletal and dentoalveolar changes were evaluated using cone beam computed tomography (CBCT) images acquired before and after expansion. Changes of the occlusal planes were observed in 10 cases (37%). Maxillary canines tended to show symmetric buccal inclinations relative to the maxillary basal bone. Six patients; 22.22% showed hypertrophy/hyperplasia of the palatal mucosa associated with ulcerations, erythema, itching, and discomfort in the area. Swelling at the mid-palatal suture after split was observed in all cases and was caused by the resultant force. No cases of necrosis of the palatal mucosa were observed. Although occlusal modifications occur after palatal split, especially in unilateral cross-bite cases, these changes can be treated with the help of fixed orthodontic appliances.

**Abstract:**

The use of maxillary expanders has the effect of distancing the maxillary bones at the level of the median palatal suture. During maxillary expansion, the main resistance forces occur at the zygomatico-maxillary sutures, and not in the median palatal suture, which is the basic principle on which this method is based. In this observational study, we evaluated possible complications at the skeletal and dentoalveolar level after palatal split using miniscrew-assisted rapid palatal expansion (MARPE) associated or not with corticopuncture (CP) therapy. The study included 27 patients with maxillary transverse deficiency and unilateral or bilateral cross-bite. Skeletal and dentoalveolar changes were evaluated using cone beam computed tomography (CBCT) images acquired before and after expansion. The mid-palatal suture was separated in 88.88% of cases, buccal bone height of the alveolar crest had decreased at first molar both at oral and palatal level by approximately 2.07 mm in 40.7% of cases whilst the remaining 59.3% showed insignificant bone loss, with canines exhibiting buccal tipping of 4.10° in 62.5% of cases. Changes of the occlusal planes were observed in 10 cases (37%). Maxillary canines tended to show symmetric buccal inclinations relative to the maxillary basal bone. Six patients; 22.22% showed hypertrophy/hyperplasia of the palatal mucosa associated with ulcerations, erythema, itching, and discomfort in the area. Swelling at the mid-palatal suture after split was observed in all cases and was caused by the resultant force. No cases of necrosis of the palatal mucosa were observed. Although occlusal modifications occur after palatal split, especially in unilateral cross-bite cases, these changes can be treated with the help of fixed orthodontic appliances.

## 1. Introduction

The miniscrew-assisted rapid palatal expansion (MARPE) was first introduced in clinical practice in 2010 [1]. The use of maxillary expanders has the effect of distancing the maxillary bones at the level of the median palatal suture [2]. During maxillary expansion, the main resistance forces occur at the zygomatico-maxillary sutures, and not in the median palatal suture, which is the basic principle on which this method is based [3]. By introducing the use of micro-screws at the level of the hard palate, an attempt was made to reduce the complications that appeared after traditional procedures of rapid maxillary expansion such as limited skeletal expansion, failure in expansion, pain, tissue swelling, buccal inclination of the supporting teeth, gingival retraction, bone resorption, and relapse [4,5] by shortening the duration of treatment, which is a goal in orthodontics [6]. The use of orthodontic micro-implants as auxiliary anchorage devices to optimize the application of mechanical forces to circum-maxillary sutures, makes it possible to avoid the otherwise indispensable osteotomies. This system, called MARPE, applies forces to the micro-implants, and not to the teeth or periodontium. The different appliance designs, conformation and techniques described in the literature, each lead to specific outcomes. In practice, clinicians have recently introduced several minimal invasive surgical techniques associated with MARPE, called corticopunctures or micro-osteoperforations, [7,8,9,10,11] to improve or facilitate the rate of tooth movement (RTM) and to reduce iatrogenic damage caused by the long-term wear of fixed appliances [12,13].

In order to achieve skeletal splitting of the mid-palatal suture, the MARPE device has to transmit strong forces to the maxilla and the anchored teeth, which are constrained from moving due to the elasticity of the periodontal ligament. Dental inclination and bending of the alveolar bone are common and unavoidable because a pure skeletal opening is not attainable. Other unfavorable effects such as root resorption, marginal bone loss, and reduction of buccal bone thickness were found in the anchoring teeth and supporting tissue [14,15,16,17].

Recently, Angelieri et al. [18] have proposed methods to stage skeletal maturation of the mid-palatal suture, since it is believed that the degree of the skeletal or dentoalveolar effect of the maxillary expansion procedure may be correlated to the maturation of the mid-palatal suture and rigid interdigitations (progressive calcification) of the mid-palatal suture, making it more resistant to split as age progresses [19,20].

Orthopantomogram or occlusal radiographs are adequate for assessing the opening of the mid-palatal suture. However, tooth movement, anatomical markings, bone anatomy and pathology are barely identifiable on these conventional two-dimensional radiographs. Cone beam computed tomography (CBCT) allows better imaging at low radiation dosages and presents a clear view of bony structures, tooth position, and type of palatal suture fusion with minimal image distortion.

Although MARPE and minimally invasive surgical techniques are often used in orthodontic clinical practice, only a few studies have been published in the literature on large groups of patients that present possible complications that may occur after practicing this method of orthodontic treatment [21,22]. In the present study, we evaluated possible complications of adult patients with maxillary compression who underwent orthodontic MARPE treatment associated or not with CP therapy.

## 2. Material and Method

In this observational study, we analyzed the various complications that could arise as part of the healing process following the placement of miniscrew-assisted rapid palatal expander (MARPE). This placement was paired with corticopuncture (CP) therapy in a subset of patients.

Patients underwent cone beam computed tomography (CBCT) examination before the treatment. The goal of the CBCT examination was to establish various anatomical markings, the degree of suture maturation, and to get a better understanding of the bone anatomy. The device used for image acquisition was a ProMax 3D CBCT. The intensity of the current was 6 mA and the tube voltage was 89 kV. The height and diameter of the cylindrical field of view was 82 mm and the voxel size was 0.2 × 0.2 × 0.2. The image format for compressing the image files was JPEG. 

The software used was OnDemand 3D data App^TM^ developed by CiberMed, Seoul, South Korea. It was used to locate possible changes at the bone level, tooth movement, and inclination or regarding palatal suture split. Our main interest lied in a volume of bone that was situated within the palatal median area. The possible modifications were analyzed using the software configuration that was set to coronal view. The results were later analyzed using Microsoft Office Excel^TM^, 2017 edition.

The following criteria were used for treatment:

- Transverse maxillary deficiency;
- Stage D or E palatal fusion (as seen in Angelieri et al. [18]) confirmed on CBCT prior to MARPE insertion;
- A medical history without orthodontic treatment;
- Crossbite (unilateral or bilateral).

The study involved twenty-seven patients with ages ranging from 19–35 years old. The median age of the patients was 24 years. The study group contained nine males and eighteen females. The patients were further divided into two separate groups.

**Figure 1:**
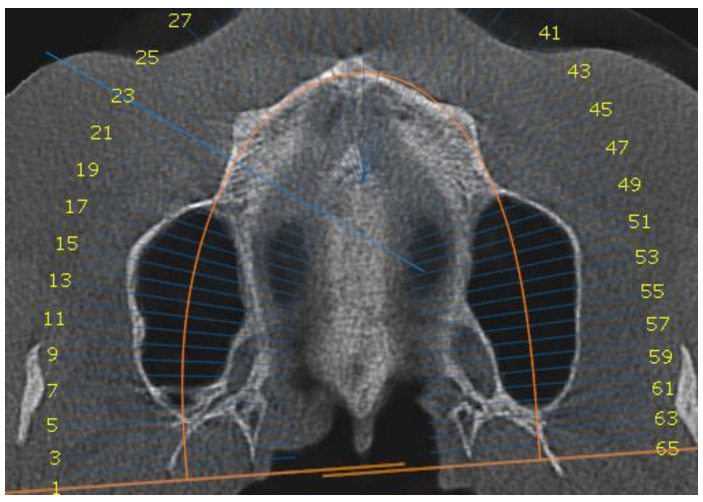
Axial view of the palatal suture. Stage E sutural fusion.

Group 1 consisted of twenty patients, thirteen females and seven males. Their age ranged from 21 to 35 years. The entire group had stage E sutural fusion. The initial observation was that the para sutural bone density did not differ from the other regions of the palate. In addition, it is important to note that the mid-palatal suture could not be identified (Figure 1).

**Figure 2:**
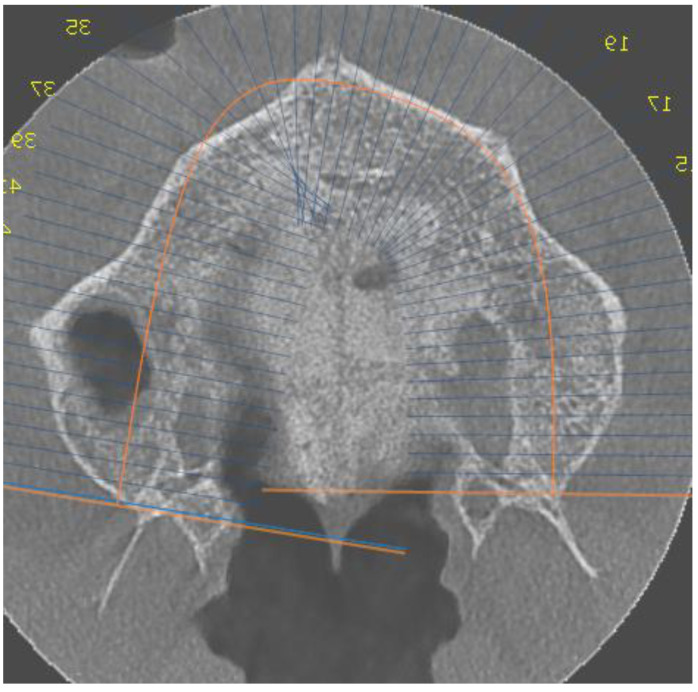
Axial view of the palatal suture. Stage D sutural fusion.

Group 2 consisted of seven patients, five females and two males, with ages ranging from 19 to 22 years. The entire group had stage D sutural fusion. The initial observation was that fusion of the mid palatal suture happened in the palatine bone. Maturation developed from posterior to anterior. Another observation was that the density of the para-sutural bone was increased when compared with the maxillary para-sutural bone (Figure 2).

The highest ethical standards were followed. They were in accordance with Helsinki Declaration of 1975, revised in 2008. They were also in accordance to the standards set by the committee for responsible human experimentation. Written informed consent was obtained from each patient that participated in this study. Furthermore, the ethics committee of the Algocalm Private Medical Center (Targu-Mures, Romania) approved the study. 

Group 1 underwent MARPE associated with CP therapy, whilst group 2 underwent conventional MARPE without CP therapy.

The following protocol was followed throughout the study:

The initial goal was to provide local anesthesia. It started with applying topical Lido-caine^TM^ Septodont (Creteil, France) 2% spray for one minute. It was followed by using a solution of epinephrine 1:100,000 (ARTICAINE^TM^ Septodont, Creteil, France) with articaine hydrochloride in order to achieve buccal infiltration of the hard palate. A gingival needle (Heraeus^TM^, Hanau, Germany) was used. It measured 0.30 × 38 mm. 

**Figure 3:**
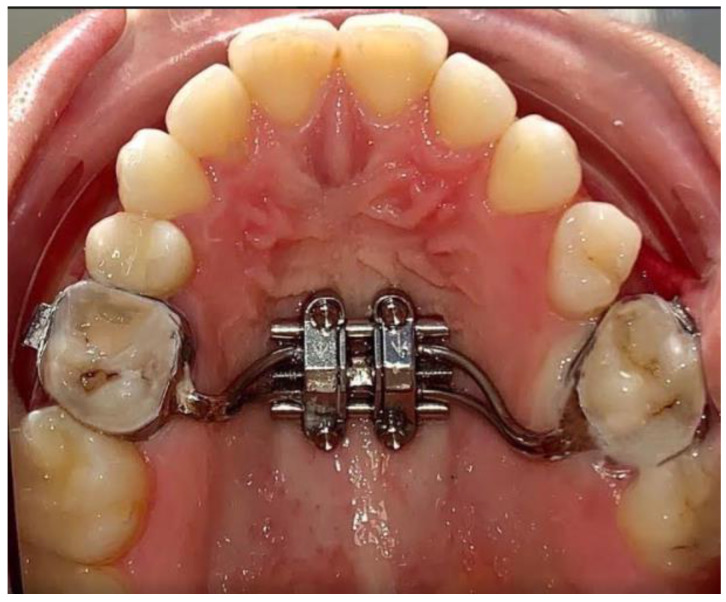
Miniscrew-assisted rapid palatal expander (MARPE) device in position without corticopuncture (CP) therapy.

The micro-screw procedure was the following:

A skeletal expander MSE II (BioMaterials^TM^, Seoul, South Korea) was applied with the use of bands around the first molar. This was followed by four mini-implants of 11 mm length and 1.8 mm diameter. The mini-implants were inserted into the palatal bone with the help of appliance slots as a rigid surgical guide (Figure 3). MSE II was positioned between first and second molars. It was situated anterior to the soft palate, but only slightly. This was done so that the expansion force can be directed against the buttress bones. A set of clinical tweezers was used to test the initial stability. The expander was activated once the stability was confirmed.

**Figure 4:**
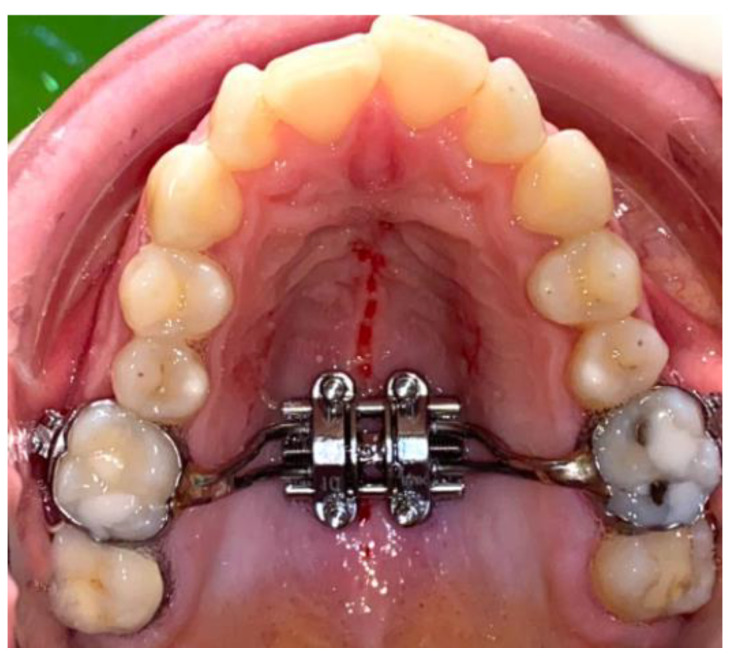
MARPE device associated with CP.

**Figure 5:**
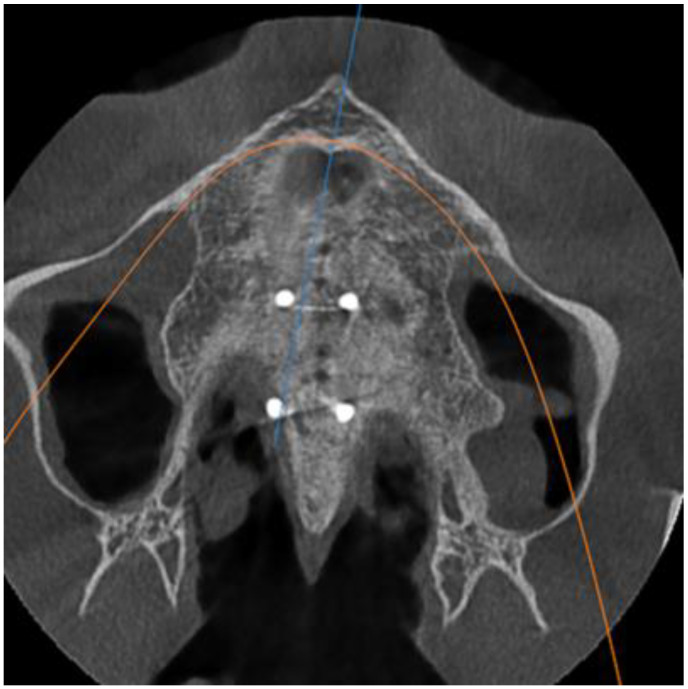
Axial view of the palatal suture showing MARPE and perforations of the palatal suture during CP.

**Figure 6:**
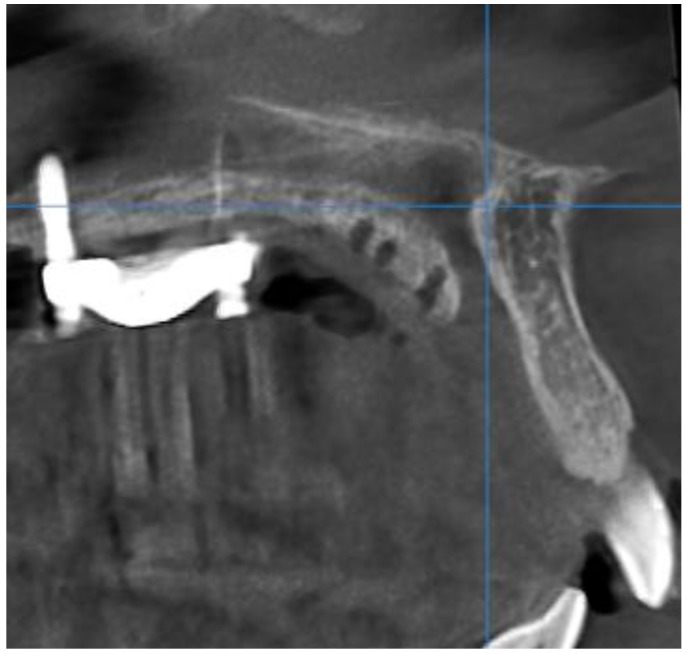
Antero-posterior view of the perforations of the palatal suture.

The following steps make up the corticopuncture (CP) therapy:

A number of bone perforations (five to ten) were done along the mid-palatal suture with the help of a round burr. The size of the round burr was 1.8 mm in diameter. Another device used was a pilot drill (MIS Implant System^TM^, Haifa, Israel) (Figure 4, Figure 5 and Figure 6).

The perforations were performed 2 mm apart. The depth ranged from 2 to 5 mm. This depended on the thickness of the cortical plate which was analyzed via a CBCT examination prior to the treatment. The minimal invasive surgical procedure was done before the insertion of MARPE device.

Antibiotics (Amoxicillin 500 mg 3 × 1/day) were prescribed after the procedure. They were paired with analgesic medication (Ibuprofen 400 mg 3 × 1/day). Another recommendation was the use of 0.12% chlorhexidine oral rinse for two weeks.

**Figure 7:**
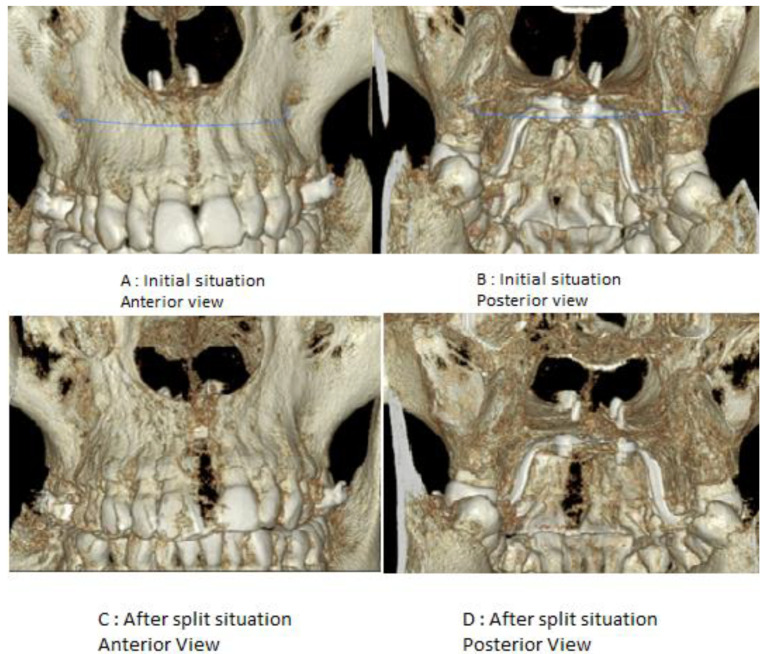
Initial situation vs after palatal suture split with MARPE.

**Figure 8:**
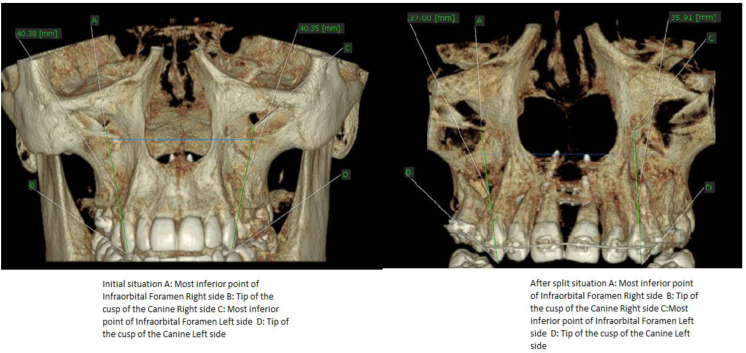
Different measurements in relation to the infraorbital foramen before and after palatal suture split with MARPE associated with CP therapy.

**Figure 9:**
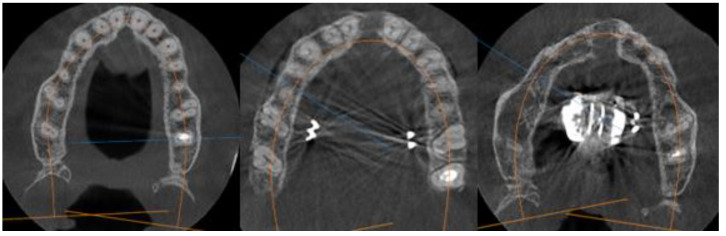
Cross sectional CBCT images before and after palatal split with MARPE device.

After the application of the MARPE device, the patients were instructed on how to perform second activation protocol, for MSE II: Minimum 4~6 turns /day (0.53~0.80 mm/day) until a diastema between the central incisors was observed (Figure 7, Figure 8 and Figure 9), indicating success in splitting the mid-palatal suture, and after the diastema appeared: 2 turns /day (0.27 mm/day) until crossbite overcorrection had been achieved. After the expansion, the MSE II remained inactivated for at least 2 months to stabilize the split. 

### 2.1. Outcome Measures

Two experienced orthodontics specialists followed up after a couple of months, performing CBCT examinations in a blind manner with the goal of assessing any complications that might have arisen (Figure 7, Figure 8 and Figure 9).

Because the evaluation of the infraorbital foramen in cone beam computed tomography (CBCT) is more accurate and reproductible, we chose this anatomical marking for the measurements involving canine tipping. The images were measured in coronal view before and after split with MARPE.

**Figure 10:**
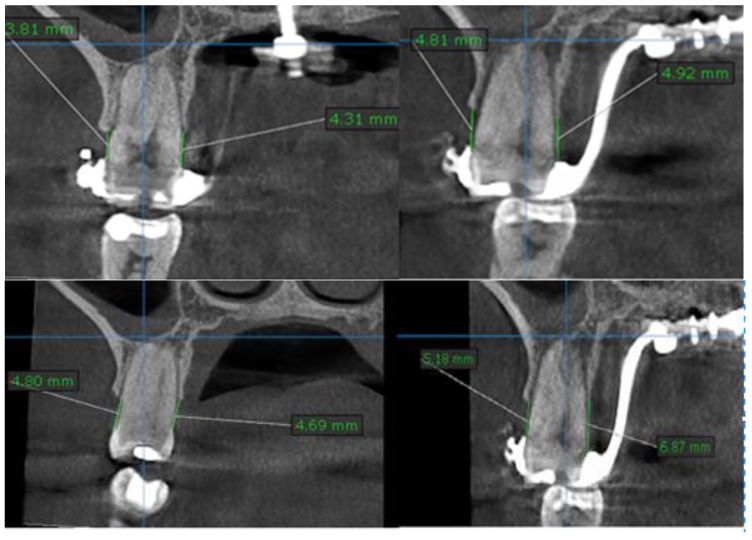
Measurements of the bone level in relation to cement-enamel junction before (**left**) and after (**right**) palatal suture split with MARPE.

In order to assess possible bone changes related to MARPE treatment, alveolar bone level measurements, prior to and after palatal split, were performed. Measurements were done from cemento-enamel junction, because this anatomical marking is stable during tooth movement, to the highest alveolar bone level, both buccal and palatal (Figure 10).

**Figure 11:**
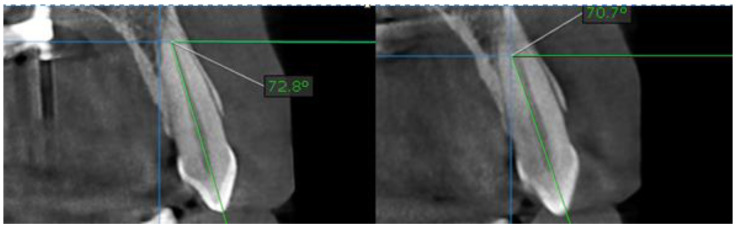
Measurements of the canine position before (**left**) and after (**right**) palatal split with MARPE.

In order to determine the type of movement that might have occurred at canine position, a line parallel to the long axis of the tooth was drawn. This was done with the aid of OnDemand 3D data App^TM^ software. The measurement was focused on identifying the angle between the horizontal plane and the drawn line. Initial situation and after split situation were compared (Figure 11).

### 2.2. Clinical Outcomes

**Figure 12:**
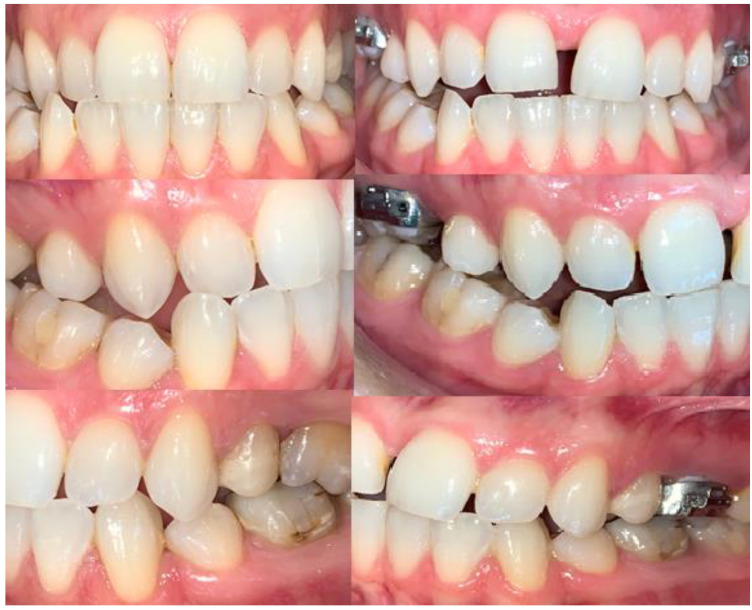
Occlusal relations before split and after.

**Figure 13:**
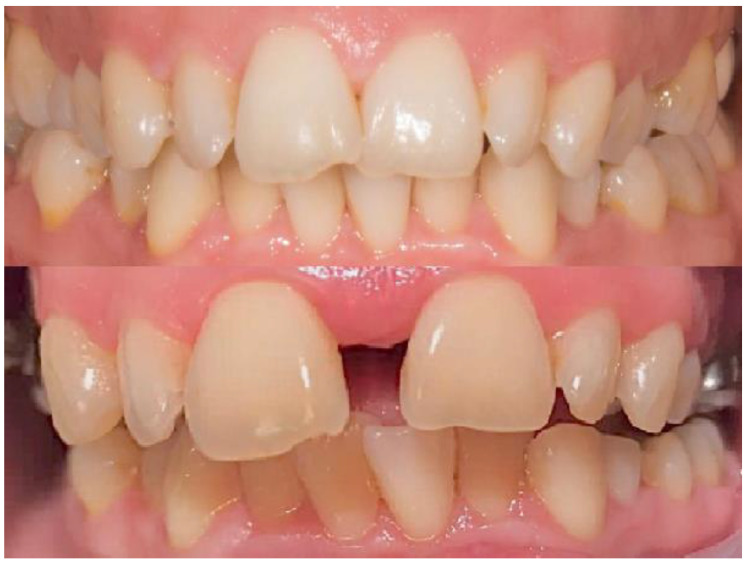
Occlusal relations before and after palatal suture split.

In order to assess possible occlusal changes after palatal split, both pictures and cast models were compared with the initial situation (Figure 12 and Figure 13).

**Figure 14:**
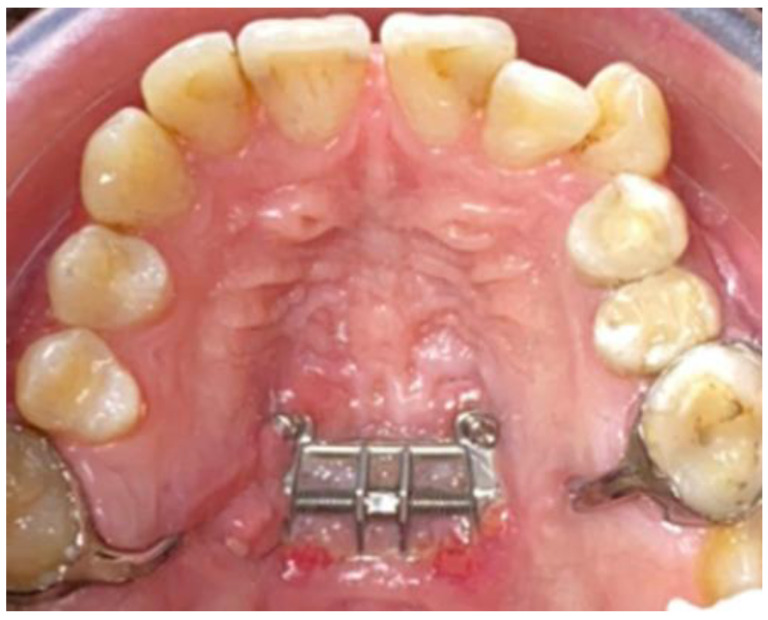
Gingival hypertrophy of the palatal mucosa associated with the MARPE device.

At a later point in the study, possible soft tissues changes related to the MARPE device were also evaluated (Figure 14).

### 2.3. Statistical Analysis

The software used for doing the statistical analysis was GraphPad Prism V.6.01. D’Agostino and Pearson normality test was performed, a versatile and powerful normality test. The goal was to identify how far the distribution was from Gaussian distribution in terms of symmetry and shape. The analysis involved also the use of the Student’s t-test for independent and dependent data. The pre-expansion values of all considered parameters were equal to zero, which suggested non-normal distribution. This led to a non-parametric test usage, alpha = 0.05 was the significance threshold chosen, determining *p* significant when *p* < 0.05.

## 3. Results

Although miniscrew-assisted rapid palatal expansion (MARPE) effectively achieved skeletal and dentoalveolar expansion by separation of the mid-palatal suture, complications related to treatment were also evident. Among the 27 patients treated by MARPE, three exhibited failure of opening of the mid-palatal suture, resulting in a success rate of 88.88%.

**Figure 15:**
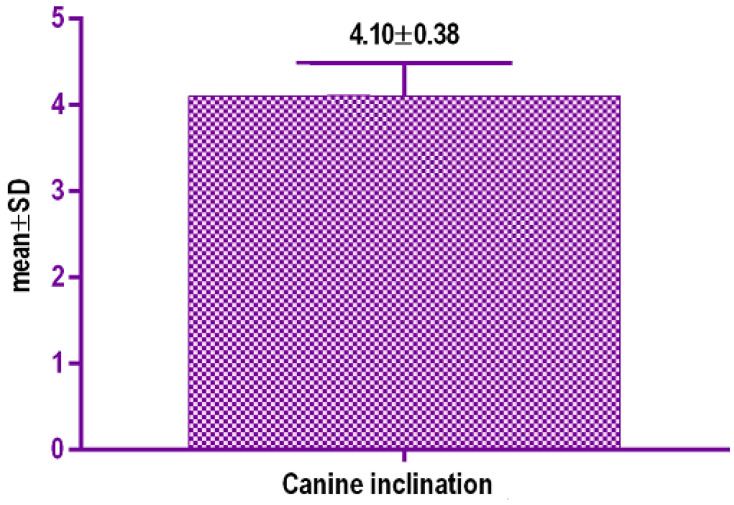
Canine tipping after split (°).

**Figure 16:**
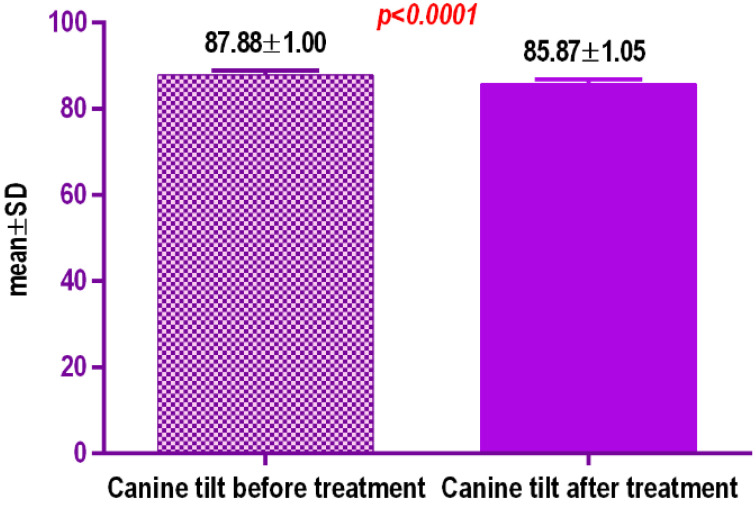
Canine tipping before and after split (°).

Evaluation of coronal images of the canines showed statistically significant (*p* < 0.001) buccal tipping of the canines in fifteen cases (62.5%), with a mean value of inclination of 4.10°+/−0.38 SD (95% CI) (Table 1 and Table 2, Figure 15 and Figure 16). Changes of the occlusal plane were observed in ten cases (37%).

**Figure 17:**
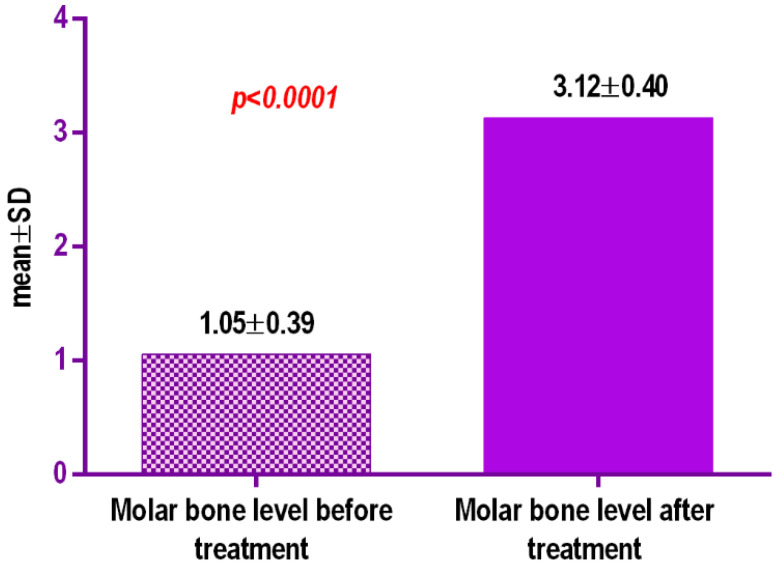
Comparative bone level before and after split (mm).

At the first molar level, a decrease in the buccal and palatal bone level was observed in eleven patients (40.70%) with a mean value of 2.07 mm+/−0.40 SD (95% CI) (Table 3, Figure 17). There was also a decrease in bone thickness associated with periodontal space enlargement. 

**Figure 18:**
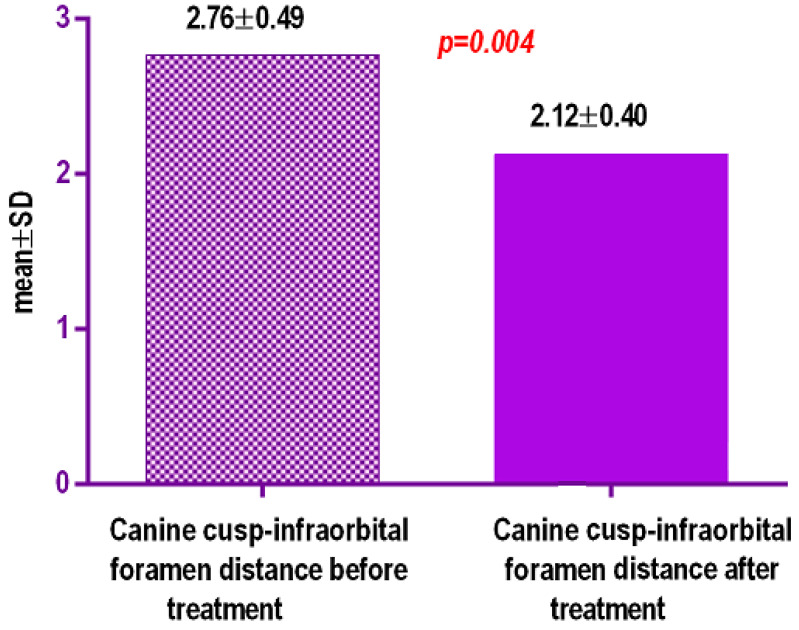
Inter-side differences in vertical distances from tip of the cusp of the canine and infraorbital foramen (mm).

Inter-side differences in tooth inclinations and vertical distances from tip of the cusp of the canine and infraorbital foramen were smaller (Table 4, Figure 18) 0.64 mm+/−0.19 SD (95% CI) *p* = 0.004, after treatment, thus confirming the tipping of the canines.

Regarding soft tissue changes related to the MARPE device, six patients (22.22%) showed hypertrophy of the palatal mucosa associated with ulcerations, erythema, itching, and discomfort in the area. No cases of necrosis of the palatal mucosa were observed. 

## 4. Discussions

The main complications of orthodontic treatment in young adult patients are gingival retraction and reduced alveolar bone thickness. Therefore, some authors recommend that surgical treatment (surgically-assisted rapid palatal expansion) should be performed in young adult patients with transverse maxillary deficiencies [23]. On the other hand, there are authors who suggest that with the introduction of new generations of microimplants into clinical practice the success rate of miniscrew-assisted rapid palatal expansion (MARPE) is greater than 80% even in young adults, with stable clinical results over time. In contrast, the results of these studies were not validated in large groups of patients [24]. However, the surgical approach in these cases is not always used at present time due to the postoperative complications that may occur (hematomas, infections, maxillary sinus trauma, maxillary nerve branch injuries, pain). It is also important to note that most patients prefer an orthodontic procedure compared to a surgical one that has a much higher morbidity rate [1]. On the other hand, there are authors who argue that with the distancing of the two maxillary bones from each other, there is a forward or posterior displacement of the two maxillary bones [25]. This could partly explain the changes in the occlusal plane observed in our study.

The main factor that can affect the prognosis of patients undergoing MARPE is represented by the multitude of sutures existing in the facial skeleton, as well as the strength of the bones at this level [26]. In this respect, a very important role is played by the junction between the palatine bones and the sphenoid bone, respectively with the 2 maxillary bones [27]. The articulation of the pyramidal process of the palatine bone with the pterygoid process of the sphenoid bone also plays a particularly important role in these cases [28]. Thus, some authors observed that after practicing MARPE the pyramidal process of the palatine bone detaches from the pterygoid process of the sphenoid bone in only 53% of cases. This is due either to the increased size of the pyramidal process or to the increased resistance. At the same time, one of the main advantages of practicing MARPE for patients with transverse maxillary deficiencies is that the spacing of the two maxillary bones occurs in parallel to the mid-palatine suture due to the dissolution of the pterygopalatine suture [29,30]. 

Other possible causes of MARPE therapeutic failure are ossification of the median palatal suture or interdigitations at the level of this suture [31]. Often, this ossification process, respectively the existence of interdigitations at the level of the mid-palatal suture are difficult to evaluate with the help of usual imaging methods. It is now known that this ossification process usually begins in the posterior part of this suture, and can often be highlighted only on the basis of histological examinations. This means that it is often impossible to evaluate the existence of ossification at the level of the mid-palatal suture before MARPE, or at the level of the other sutures of the facial skeleton [15,28,29]. The possible existence of foci of ossification, strong piriform aperture pillars and zygomatic buttresses could be one of the causes that led to the failure of MARPE in three of the patients in our study. Other causes that can lead to therapeutic failure in such cases may be the possible occurrence of complications in the palatal mucosa (mucosal ischemia or necrosis, local infections) [32,33].

Given the possibility of therapeutic failure, due in part to the increased resistance of the mid-palatal suture, some authors recommend performing small punctures, so-called corticopunctures, in the cortex of the palatine bone corresponding to the mid-palatal suture in order to reduce bone strength at this level [30]. The practice of these small osteo-perforations in the palatine bone creates the premises for the migration of cytokines to these sites, which leads to the activation of osteoclasts, which in turn will facilitate the process of bone remodeling [2,3]. On rabbits, looking at maxillary expansion, Pulver et al. suggested that greater skeletal expansions may be possible when combined with surgical methods, such as CP, as this method promotes regional acceleratory phenomenon, reducing bone volume and density and stimulating bone remodeling [34]. Suzuki et al. demonstrated that, when performed along the mid-palatal suture, a minimally invasive surgical method such as CP permitted suture split and accelerated bone remodeling when this failed to occur after the conventional protocol for MARPE activation [8].

Some authors also recommend the practice of peeling at the level of the buccal and palatal walls of the alveolar bone in order to reduce possible periodontal complications at this level. However, when practicing these methods, local complications can be quite common [35]. Tsai et al. compared the effects of corticotomy and bone microperforations and concluded that both techniques increased bone remodeling and there were no significant differences between them [36]. However, subcutaneous hematomas and postoperative swelling and discomfort were also associated with the corticotomy procedure [37].

The activation force of rapid maxillary expander (RME) device initially results in the compression of the periodontal ligament, bending of the alveolar bone, and tipping of the anchored teeth. Therefore, a 1°–24° increase in molar inclination is inevitable, probably because of alveolar bending and/or tipping of the posterior teeth. Winsauer et al. showed this in a study of 33 adults, between 23 and 33 years, where 90% of the patients had successful palatal widening without surgical assisted rapid palatal expander (SARPE) and no dental side effects [38]. The tipping of the canine treatment is conditioned by the early and correct diagnosis and an appropriate treatment planning, which often requires an interdisciplinary approach [39].

Regarding soft tissues, cases of ulceration of the upper jaw and pyogenic granuloma caused by rapid palatal expander appliances have been reported [40]. These results are consistent with our finding and may be related to poor oral hygiene around the MARPE appliance.

## 5. Conclusions

In our study, MARPE effectively achieved dentoalveolar as well as skeletal expansion by separation of the mid-palatal suture in 88.88% of cases. A decrease in bone level and thickness at first molars in 40.7% of the cases was also evident. Maxillary canines tended to show symmetric buccal inclinations relative to the maxillary basal bone. Changes of the occlusal planes were observed in 10 cases (37%). Although occlusal modifications occur after palatal split, especially in unilateral cross-bite cases, these changes can be treated with the help of fixed orthodontic appliances. Undesirable effects like discomfort at the level of the incisors or nasal area, ulcerations, oedema of the palatal mucosa were observed in 22.22% of cases. Swelling at the mid-palatal suture after split was observed in all cases and was caused by the resultant force. 

## Figures and Tables

**Table 1:** Mean values of canine tipping (°).

Data Values	Data 1
*p* value	<0.0001
*p* value summary	
Significantly different? (*p* < 0.05)	Yes
One-or two-tailed *p* value?	Two-tailed
*t*, *df*	*t* = 12.33, *df* = 19
Number of pairs	48
Mean of differences	4.10
SD of differences	0.7273
SEM of differences	0.1626
95% confidence interval	2.345 to 1.665
R square	0.8889
Correlation coefficient (r)	0.7515
*p* value (one tailed)	<0.0001
*p* value summary	
Significant correlation? (*p* > 0.05)	Yes

**Table 2:** Canine tipping after split (°).

Data	Canine Inclination/°
Mean	4.10
25% Percentile	2.783
Median	3.21
75% Percentile	3.865
Maximum	4.77
Mean	4.103
Std. Deviation	0.3881
Std. Error of Mean	0.08679
Lower 95% CI of mean	2.921
Upper 95% CI of mean	3.784

**Table 3:** Comparative bone level before and after split (mm).

	Molar Bone Level Before Treatment	Molar Bone Level after Split
Number of values	30	30
Minimum	1.97	3.67
25% Percentile	2.25	3.78
Median	2.2	3.995
75% Percentile	4.165	2.385
Maximum	4.54	6.11
Mean	1.056	3.125
Std. Deviation	0.3956	0.407
Std. Error of Mean	0.1108	0.09102
Lower 95% CI of mean	1.534	2.934
Upper 95% CI of mean	1.998	3.316

**Table 4:** Inter-side differences in vertical distances from tip of the cusp of the canine and infraorbital foramen (mm).

	Canine Cusp-Infraorbital Foramen Distance before Treatment/mm	Canine Cusp-Infraorbital Foramen Distance after Treatment/mm
Number of values	48	48
Minimum	41.97	38.67
25% Percentile	41.25	37.82
Median	41.8	39.155
75% Percentile	40.655	38.5
Maximum	42.4	41.11
Mean	2.766	2.125
Std. Deviation	0.4956	0.407
Std. Error of Mean	0.1108	0.09102
Lower 95% CI of mean	2.534	1.934
Upper 95% CI of mean	2.998	2.316

## Data Availability

Not applicable.
